# Soluble cerebral Aβ protofibrils link Aβ plaque pathology to changes in CSF Aβ_42_/Aβ_40_ ratios, neurofilament light and tau in Alzheimer’s disease model mice

**DOI:** 10.1038/s43587-025-00810-8

**Published:** 2025-02-12

**Authors:** Emelie Andersson, Nils Lindblom, Shorena Janelidze, Gemma Salvadó, Eleni Gkanatsiou, Linda Söderberg, Christer Möller, Lars Lannfelt, Junyue Ge, Jörg Hanrieder, Kaj Blennow, Tomas Deierborg, Niklas Mattsson-Carlgren, Henrik Zetterberg, Gunnar Gouras, Oskar Hansson

**Affiliations:** 1 Clinical Memory Research Unit, Lund University, Lund, Sweden; 2 Department of Experimental Medical Science, Experimental Dementia Research Unit, Lund University, Lund, Sweden; 3 BioArctic AB, Stockholm, Sweden; 4 Department of Public Health and Caring Sciences, Geriatrics, Uppsala University, Uppsala, Sweden; 5 Department of Psychiatry and Neurochemistry, Institute of Neuroscience and Physiology, The Sahlgrenska Academy at the University of Gothenburg, Mölndal, Sweden; 6 Clinical Neurochemistry Laboratory, Sahlgrenska University Hospital, Mölndal, Sweden; 7 Department of Neurodegenerative Disease, UCL Queen Square Institute of Neurology, London, UK; 8 Department of Experimental Medical Science, Experimental Neuroinflammation Laboratory, Lund University, Lund, Sweden; 9 Department of Neurology, Skåne University Hospital, Lund University, Lund, Sweden; 10 Wallenberg Center for Molecular Medicine, Lund University, Lund, Sweden; 11 UK Dementia Research Institute at UCL, London, UK; 12 Hong Kong Center for Neurodegenerative Diseases, Hong Kong, China; 13 Wisconsin Alzheimer’s Disease Research Center, University of Wisconsin School of Medicine and Public Health, University of Wisconsin-Madison, Madison, WI USA; 14 Memory Clinic, Skåne University Hospital, Malmö, Sweden

**Keywords:** Neuroscience, Biomarkers, Ageing

## Abstract

The Aβ_42_/Aβ_40_ ratio in the cerebrospinal fluid (CSF) and the concentrations of neurofilament light (NfL) and total tau (t-tau) are changed in the early stages of Alzheimer’s disease (AD)^1^, but their neurobiological correlates are not entirely understood. Here, we used 5xFAD transgenic mice to investigate the associations between these CSF biomarkers and measures of cerebral Aβ, including Aβ_42_/Aβ_40_ ratios in plaques, insoluble fibrillar deposits and soluble protofibrils. A high Aβ_42_/Aβ_40_ ratio in soluble protofibrils was the strongest independent predictor of low CSF Aβ_42_/Aβ_40_ ratios and high CSF NfL and t-tau concentrations when compared to Aβ_42_/Aβ_40_ ratios in plaques and insoluble fibrillar deposits. Furthermore, the Aβ_42_/Aβ_40_ ratio in soluble protofibrils fully mediated the associations between the corresponding ratio in plaques and all the investigated CSF biomarkers. In *App*^NL-G-F/NL-G-F^ knock-in mice, protofibrils fully mediated the association between plaques and the CSF Aβ_42_/Aβ_40_ ratio. Together, the results suggest that the Aβ_42_/Aβ_40_ ratio in CSF might better reflect brain levels of soluble Aβ protofibrils than insoluble Aβ fibrils in plaques in AD. Furthermore, elevated concentrations of NfL and t-tau in CSF might be triggered by increased brain levels of soluble Aβ protofibrils.

## Abstract (web summary)

Andersson et al. identify that high Aβ_42_/Aβ_40_ ratios in brain protofibrils best predict lower Aβ_42_/Aβ_40_ ratios in the cerebrospinal fluid (CSF) and higher neurofilament light/total tau levels, indicating that CSF changes in Alzheimer’s disease reflect soluble protofibrils more accurately than amyloid plaques.

## Main

Alzheimer’s disease (AD) is pathologically characterized by the deposition of amyloid-β (Aβ) plaques, the formation of tau-containing intracellular aggregates and neurodegeneration^2^. These events are associated with altered concentrations of cerebrospinal fluid (CSF) biomarkers, which have become important in the clinical workup of the disease and clinical trials^1^.

The CSF Aβ_42_/Aβ_40_ ratio is a well-established biomarker of Aβ pathology, which is reduced already in the preclinical stage of AD. Its decline is attributed to altered concentrations of Aβ_42_, while those of Aβ_40_ are unchanged during the disease^3^. Postmortem and in vivo Aβ positron emission tomography (PET) studies demonstrated an inverse association between CSF Aβ_42_—alone or in ratio with Aβ_40_—and the cerebral burden of Aβ plaques^4–8^. These results set the foundation for the hypothesis that reduced CSF Aβ_42_/Aβ_40_ ratios are due to the deposition of highly aggregation-prone Aβ_42_ into insoluble fibrils in Aβ plaques, resulting in lower soluble concentrations of Aβ_42_ available for transport to the CSF^9^. However, some findings indicate that this explanation may be somewhat simplified. Neuropathological evaluation of brains from deceased patients with AD revealed that Aβ_40_ also accumulates in Aβ plaques^10^; however, notably this does not affect its concentration in the CSF^11^. Furthermore, the inverse association with Aβ PET, which detects insoluble fibrillar Aβ species^12^ that dominate in the center of dense-cored plaques^2^, is characterized by CSF Aβ_42_/Aβ_40_ ratios reaching a plateau, although the load of fibrillar Aβ in the brain continues to increase^6,7^. This suggests a limited impact of the accumulation of fibrillar Aβ in the brain on CSF Aβ_42_/Aβ_40_ ratios, at least when Aβ deposition has become established. Thus, to optimize its use in clinical practice and clinical trials, further studies are needed to increase our understanding of the underlying pathological events that are reflected by altered CSF Aβ_42_/Aβ_40_ ratios.

Soluble Aβ oligomers and protofibrils are, in addition to insoluble fibrillar Aβ species, increased in the brains of patients with AD^13–16^. Interestingly, rare familial forms of AD caused by the Arctic and Osaka mutations, which specifically enhance the formation of these soluble Aβ aggregates, are characterized by low CSF Aβ_42_/Aβ_40_ ratios and Aβ_42_ concentrations despite the absence of fibrillar Aβ in the brain as visualized by Aβ PET^17,18^. These results suggest a possible link between soluble Aβ oligomers and protofibrils in the brain and lowered Aβ_42_/Aβ_40_ ratios in the CSF that has not been addressed previously. Studies on carriers of these rare mutations indicate that soluble Aβ oligomers and protofibrils are the most pathogenic forms of Aβ^19^, considering that these individuals develop early dementia in the absence of cored Aβ plaques^18,20^. Indeed, many studies showed that soluble Aβ oligomers and protofibrils impair synaptic structures and functions^13,21–25^ and cause selective neuronal death^26,27^ in different in vitro and in vivo model systems. We and others have previously shown that the concentrations of two established CSF biomarkers of neurodegeneration—neurofilament light (NfL) and total tau (t-tau)—are increased in response to early Aβ pathology in both humans^8,28–30^ and transgenic mouse models^8,30–33^. Nevertheless, the degree to which soluble Aβ oligomers and protofibrils may be associated with these changes is unclear.

In this study, we measured Aβ_42_/Aβ_40_ ratios and concentrations of NfL and t-tau in CSF collected at different time points from the 5xFAD transgenic mouse model of AD. We investigated the associations between these CSF biomarkers and measures of cerebral Aβ pathology, including Aβ_42_/Aβ_40_ ratios in plaques, insoluble formic acid-extracted Aβ and soluble protofibrils. Specifically, we examined which of these measures of cerebral Aβ pathology are independently associated with reduced Aβ_42_/Aβ_40_ ratios and increased concentrations of NfL and t-tau in the CSF.

## Results

**Fig. 1:**
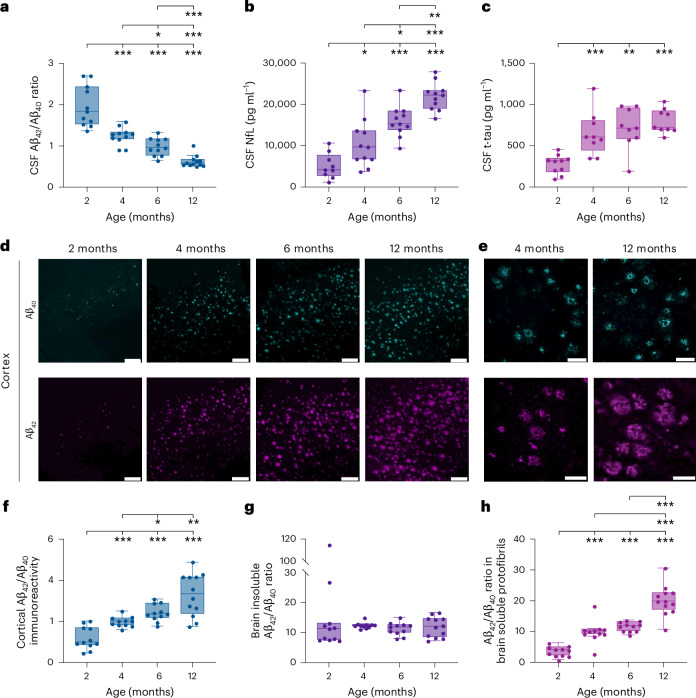
CSF biomarkers and cerebral Aβ pathology in 5xFAD mice. The Aβ_42_/Aβ_40_ ratio, NfL and t-tau in the CSF, and cerebral Aβ pathology, were measured in two (*n* = 11), four (*n* = 11), six (*n* = 11) and 12-month-old (*n* = 12) 5xFAD mice. **a**–**c**, The Aβ_42_/Aβ_40_ ratio was reduced (**a**) and the concentrations of NfL (**b**) and t-tau (**c**) in the CSF were increased in an age-dependent manner. **d**, The burden of cortical Aβ_42_ and Aβ_40_ immunoreactivity was increased in an age-dependent manner. **e**, Aβ_42_ was more evenly distributed in the plaques while Aβ_40_ was more abundant around the core. **f**, The relative cortical deposition of Aβ_42_ was higher than that for Aβ_40_, resulting in increased cortical Aβ_42_/Aβ_40_ immunoreactivity with age. **g**, Brain insoluble Aβ_42_/Aβ_40_ was not affected by age. **h**, The Aβ_42_/Aβ_40_ ratio in soluble protofibrils was substantially increased in an age-dependent manner. Data are presented as the median and interquartile range (IQR). The whiskers represent data within 1.5 times the IQR of the lower and upper quartiles. For comparisons between groups, statistical analysis was performed using a Kruskal–Wallis test followed by a two-tailed Mann–Whitney *U*-test for post hoc group comparisons. *P < 0.05, **P < 0.01, ***P < 0.001. The exact *P* values are reported in the source data file for Fig. 1. No adjustments were made for multiple comparisons. **d**, Scale bar, 200 μm. **e**, Scale bar, 50 μm. Source data

**Extended Data Fig. 1:**
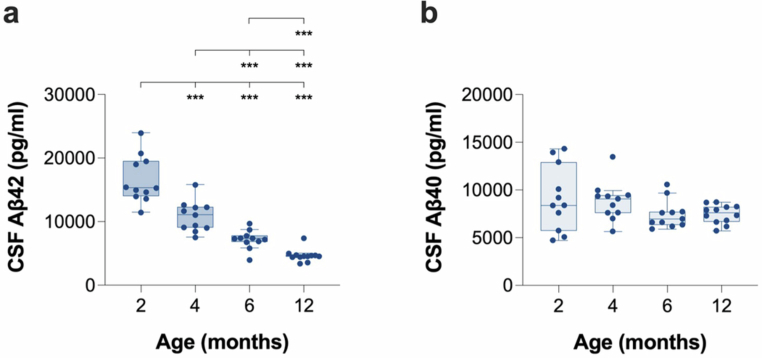
CSF Aβ42 and Aβ40 in 5xFAD mice. CSF Aβ42 and Aβ40 were measured in 2 (*n* = 11), 4 (*n* = 11), 6 (*n* = 11), and 12 (*n* = 12) months old 5xFAD mice. (**a**) The concentration of CSF Aβ42 was reduced in an age-dependent manner while (**b**) Aβ40 remained unchanged. Data are presented as median and IQR. Whiskers represent data within 1.5IQR of the lower and upper quartiles. For comparison between groups, statistical analyses were perform using the Kruskal-Wallis H test followed by the two-tailed Mann-Whitney U test for post hoc group comparisons (***p < 0.001). The exact p-values are reported in the Source Data File for Extended Data Fig. 1. The results were not adjusted for multiple comparisons. Abbreviations: Aβ, amyloid beta; CSF, cerebrospinal fluid; IQR, interquartile range. Source data

In agreement with what has previously been reported in other *APP*-overexpressing mouse models of AD^32,34^, we found that the Aβ_42_/Aβ_40_ ratio in the CSF from 5xFAD mice was affected by age (*H*(3) = 36.3, *P* < 0.001) (Fig. 1a and Supplementary Table 1). At 4 months, the Aβ_42_/Aβ_40_ ratio in the CSF was reduced by 30% compared to the youngest group; at 12 months, a 68% decline was observed. The age-dependent change in the Aβ_42_/Aβ_40_ ratio was due to reduced concentrations of CSF Aβ_42_ over time (*H*(3) = 37.0, *P* < 0.001), while Aβ_40_ was unaffected (*H*(3) = 4.8, *P* > 0.05) (Extended Data Fig. 1a,b).

**Extended Data Fig. 2:**
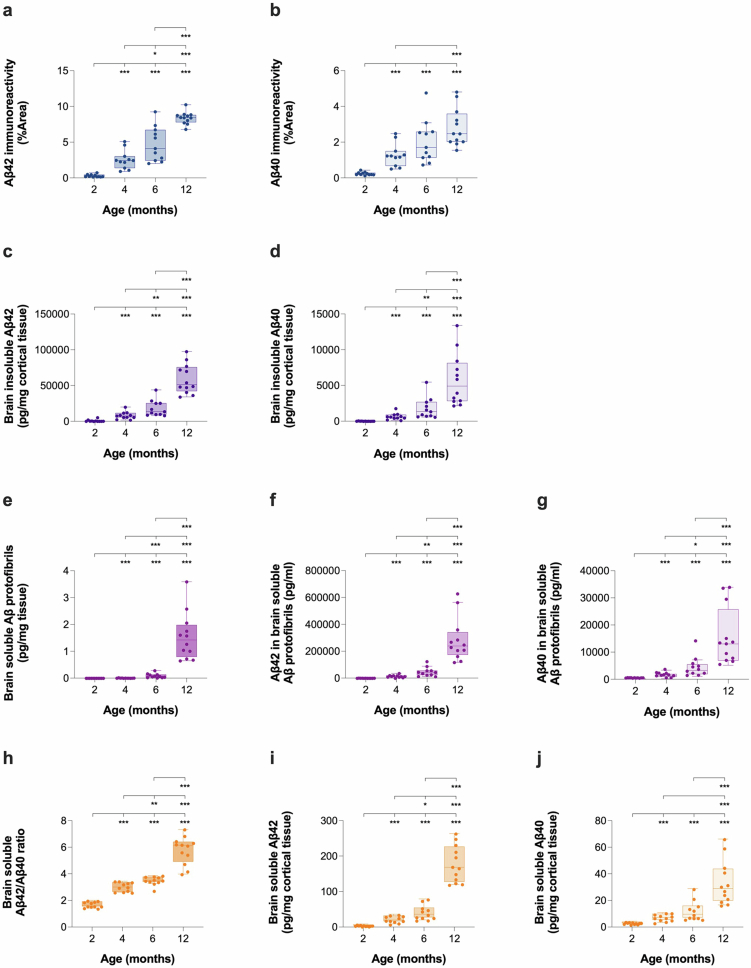
Cerebral measures of Aβ42 and Aβ40 in 5xFAD mice. Cerebral Aβ42 and Aβ40 were measured in 2 (*n* = 11), 4 (*n* = 11), 6 (*n* = 11), and 12 (*n* = 12) months old 5xFAD mice. (**a**-**b**) Cortical Aβ42 and Aβ40 immunoreactivity were increased in an age-dependent manner. (**c**-**d**) Age did also affect the concentrations of Aβ42 and Aβ40 in insoluble fibrillar deposits, (**e**) the concentration of soluble Aβ protofibrils, (**f**-**g**) the concentrations of Aβ42 and Aβ40 in soluble protofibrils, and (**h**-**j**) the total concentration of soluble Aβ42 and Aβ40 within the brains of 5xFAD mice. Data are presented as median and IQR. Whiskers represent data within 1.5IQR of the lower and upper quartiles. For comparison between groups, statistical analyses were perform using the Kruskal-Wallis H test followed by the two-tailed Mann-Whitney U test for post hoc group comparisons (*p < 0.05, **p < 0.01, ***p < 0.001). The exact p-values are reported in the Source Data File for Extended Data Fig. 2. The results were not adjusted for multiple comparisons. Abbreviations: Aβ, amyloid beta; CSF, cerebrospinal fluid; IQR, interquartile range. Source data

**Extended Data Fig. 3:**
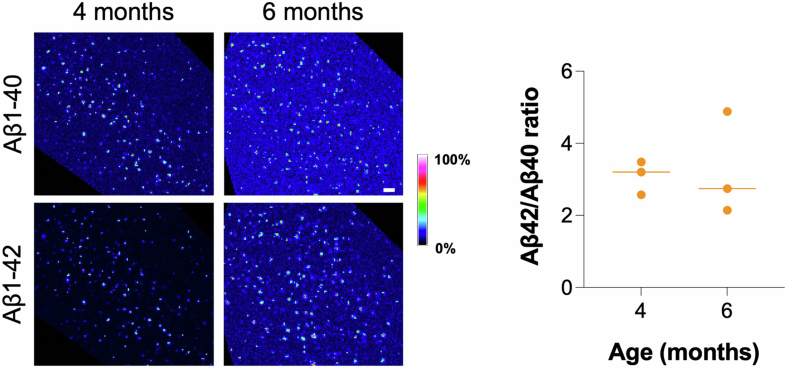
The Aβ1-42/Aβ1-40 ratio in cortical extracellular plaques in 5xFAD mice. The deposition of Aβ1-40 and Aβ1-42 in cortical extracellular plaques was assessed in 4 (*n* = 3)- and 6 (*n* = 3)-months-old 5xFAD mice using label-free MALDI MSI. **(a)** Signal ion images of Aβ1-40 and Aβ1-42 present in extracellular plaques captured in the frontal cortex of 4- and 6-months-old 5xFAD mice. **(b)** Assessment of the Aβ1-42/Aβ1-40 signal intensity ratio in extracellular plaques captured in the frontal cortex of 4- and 6-months-old 5xFAD mice showed that the deposition of Aβ1-42 was higher compared with Aβ1-40 at the two investigated time points. Number of plaques analyzed for each animal: 4-6 plaques. Scale bar: 100 μm. Abbreviations: Aβ, amyloid beta; MALDI, matrix assisted laser desorption/ionization; MSI, mass spectrometry imaging.

Further analysis revealed that the decline of the CSF Aβ_42_/Aβ_40_ ratio coincided with an age-dependent increase in cortical Aβ_42_ and Aβ_40_, as determined using immunohistochemistry (Fig. 1d). Notably, while Aβ_42_ was more evenly distributed in the plaques as the mice aged, Aβ_40_ was particularly abundant around the core (Fig. 1e). Moreover, the relative cortical deposition of Aβ_42_ was higher than that of Aβ_40_, resulting in increased Aβ_42_/Aβ_40_ ratios with age (Fig. 1f and Extended Data Fig. 2a,b). In a subgroup of 5xFAD mice, similar Aβ_42_/Aβ_40_ ratios in cortical plaques were found using label-free matrix-assisted laser desorption/ionization mass spectrometry imaging (Supplementary Methods and Extended Data Fig. 3).

Our observation that Aβ_40_ deposits in plaques without affecting its concentrations in the CSF aligns with findings in patients with AD^10,11^. Under physiological conditions, the concentration of Aβ_40_ in the CSF is about ten times higher than Aβ_42_. This led to speculation that the accumulation of Aβ_40_ in extracellular deposits is too low to induce measurable changes in the CSF^35^. However, in 5xFAD mice, where the production of Aβ_42_ is favored over Aβ_40_ (ref. ^36^), no temporal changes in CSF Aβ_40_ were evident despite substantial Aβ_40_ immunoreactivity in the brain. These results challenge the current hypothesis linking the decline of Aβ peptides in the CSF to their relative deposition in plaques and indicate that the underlying cause according to which the CSF Aβ_42_/Aβ_40_ ratio is reduced in AD might be due to more complex mechanisms.

Insoluble fibrillar Aβ found predominantly in the core of plaques can be measured biochemically in formic acid extract from brain homogenates. In 5xFAD mice, we did not observe an effect of age on the Aβ_42_/Aβ_40_ ratio in insoluble brain extracts (*H*(3) = 2.1, *P* > 0.05). Instead, the concentration of Aβ_42_ remained about 12 times higher than that of Aβ_40_ over time (Fig. 1g and Extended Data Fig. 2c,d). We also measured the concentration of soluble Aβ protofibrils and their Aβ_42_ and Aβ_40_ content in cortical brain tissue from 5xFAD mice. The concentration of soluble Aβ protofibrils was increased over time (*H*(3) = 41.1, *P* < 0.001) (Extended Data Fig. 2e), as well as the Aβ_42_/Aβ_40_ ratio in these aggregates (*H*(3) = 35.0, *P* < 0.001). Notably, at 12 months, the concentration of Aβ_42_ in soluble Aβ protofibrils was about 20 times higher than that of Aβ_40_ (Fig. 1h and Extended Data Fig. 2f,g). The increased concentration of soluble Aβ protofibrils corresponded with an elevated overall concentration of soluble Aβ_42_ and Aβ_40_ in the brain and an increased Aβ_42_/Aβ_40_ ratio (Extended Data Fig. 2h–j).

### The Aβ_42_/Aβ_40_ ratio in brain soluble protofibrils most accurately predicts the drop in the CSF Aβ_42_/Aβ_40_ ratio

**Fig. 2:**
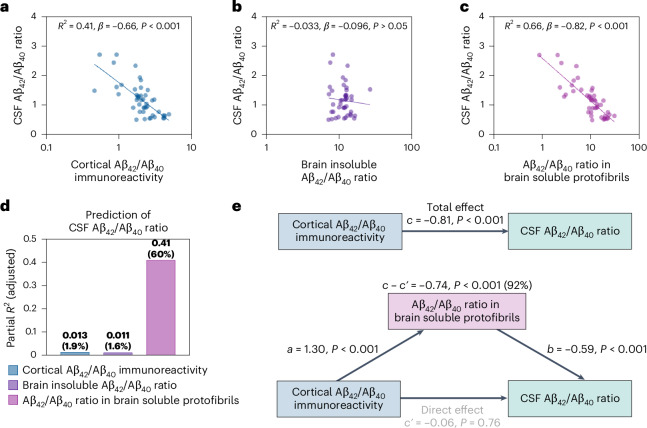
Associations between the CSF Aβ_42_/Aβ_40_ ratio and measures of cerebral Aβ pathology. **a**–**c**, In simple linear regression models, the CSF Aβ_42_/Aβ_40_ ratio was inversely associated with cortical Aβ_42_/Aβ_40_ immunoreactivity (**a**) and the Aβ_42_/Aβ_40_ ratio in brain soluble protofibrils (**c**), while no association with the brain insoluble Aβ_42_/Aβ_40_ ratio was found (**b**). **d**, In the multiple linear regression model, lower CSF Aβ_42_/Aβ_40_ ratios were independently associated with higher Aβ_42_/Aβ_40_ ratios in soluble protofibrils and, to a very minor extent, lower brain insoluble Aβ_42_/Aβ_40_ ratios. The bars represent the partial *R*^2^ for each predictor in the model. The percentage within the parentheses represents the ratio between the partial *R*^2^ and the total *R*^2^ of the model. **e**, Mediation analysis revealed that 92% of the direct effect of cortical Aβ_42_/Aβ_40_ immunoreactivity on the CSF Aβ_42_/Aβ_40_ ratio was explained by the Aβ_42_/Aβ_40_ ratio in soluble protofibrils. Mediation analysis alone does not establish causation. All analyses were adjusted for sex. The exact *P* values are reported in the source data file for Fig. 2. Source data

We next investigated the associations between the Aβ_42_/Aβ_40_ ratio in the CSF and the different measures of cerebral Aβ pathology. Simple linear regression models adjusted for sex showed that the CSF Aβ_42_/Aβ_40_ ratio was inversely correlated with cortical Aβ_42_/Aβ_40_ immunoreactivity (*R*^2^ = 0.41, *β* = −0.66, *P* < 0.001) (Fig. 2a) and soluble Aβ_42_/Aβ_40_ protofibrils (*R*^2^ = 0.66, *β* = −0.82, *P* < 0.001) (Fig. 2c), while no correlation was found with the Aβ_42_/Aβ_40_ ratio in formic acid-extracted Aβ (*R*^2^ = −0.033, *β* = −0.096, *P* > 0.05) (Fig. 2b). Comparison of these models revealed that soluble Aβ_42_/Aβ_40_ protofibrils explained more of the variance in the outcome (CSF Aβ_42_/Aβ_40_) than cortical Aβ_42_/Aβ_40_ immunoreactivity (Δ*R* = 0.25, 95% confidence interval (CI) = 0.085–0.46, *P* < 0.01).

To examine the independent associations between the specific measures of Aβ pathology and reduced Aβ_42_/Aβ_40_ ratios, we further built a multiple linear regression model. In this model, all three measures of Aβ pathology were included as predictors of the CSF Aβ_42_/Aβ_40_ ratio. The CSF Aβ_42_/Aβ_40_ ratio showed a negative association with soluble Aβ_42_/Aβ_40_ protofibrils (*β* = −0.76, *P* < 0.001) and a positive association with the Aβ_42_/Aβ_40_ ratio in formic acid-extracted Aβ (*β* = 0.20, *P* < 0.05). The association with cortical Aβ_42_/Aβ_40_ immunoreactivity was nonsignificant (*β* = −0.12, *P* = 0.49). The soluble Aβ_42_/Aβ_40_ protofibrils explained the maximum percentage of the variance in the CSF Aβ_42_/Aβ_40_ ratio (partial *R*^2^ = 0.41, percentual *R*^2^ = 60%). This was notably higher than that explained by the Aβ_42_/Aβ_40_ ratio in formic acid-extracted Aβ (partial *R*^2^ = 0.011, percentual *R*^2^ = 1.6%) (Fig. 2d). When additionally including the overall brain soluble Aβ_42_/Aβ_40_ ratio in the model, the Aβ_42_/Aβ_40_ ratio in soluble protofibrils was the strongest independent predictor of low CSF Aβ_42_/Aβ_40_ ratios (Supplementary Table 2).

**Extended Data Fig. 4:**
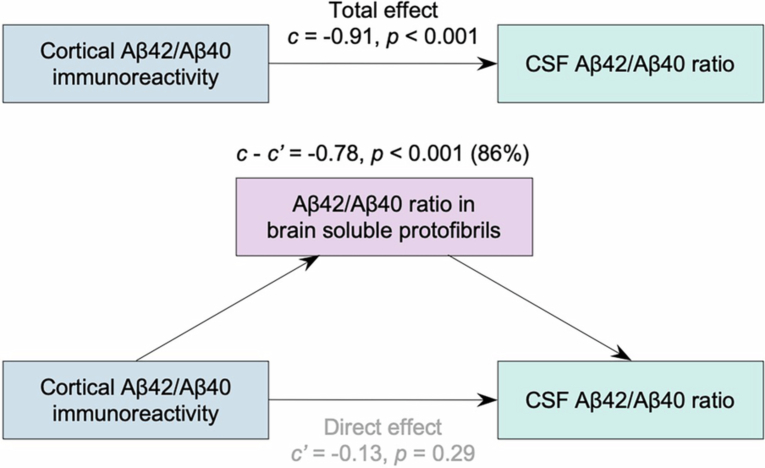
Mediation effect of soluble Aβ42/Aβ40 protofibrils in 5xFAD mice. Soluble Aβ42/Aβ40 protofibrils fully mediated the effect of cortical Aβ42/Aβ40 immunoreactivity on the CSF Aβ42/Aβ40 ratio when the models were adjusted for sex and brain insoluble Aβ42/Aβ40 ratios. Mediation analysis alone does not establish causation. Abbreviations: Aβ, amyloid beta; CSF, cerebrospinal fluid.

As the negative association between cortical Aβ_42_/Aβ_40_ immunoreactivity and the corresponding CSF ratio was lost when moving from the univariate to the multivariate analysis, we examined if the association between these two variables was statistically mediated by the Aβ_42_/Aβ_40_ ratio in soluble protofibrils. Indeed, statistical mediation analysis adjusted for sex revealed that soluble Aβ_42_/Aβ_40_ protofibrils fully mediated the effect of cortical Aβ_42_/Aβ_40_ immunoreactivity on the Aβ_42_/Aβ_40_ ratio in the CSF (percentage mediation = 92%) (Fig. 2e). Similar results were obtained when the model was additionally adjusted for the Aβ_42_/Aβ_40_ ratio in formic acid-extracted Aβ (Extended Data Fig. 4).

**Extended Data Fig. 5:**
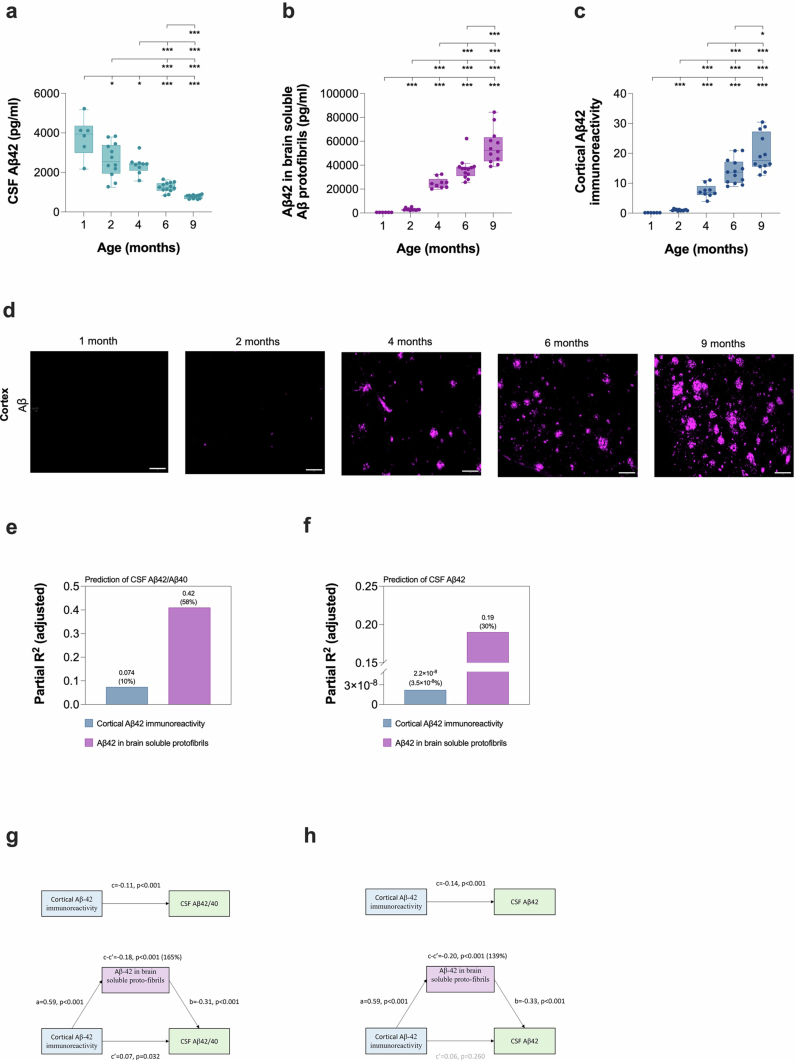
Aβ42 in CSF, soluble Aβ protofibrils, and cortical plaques in App^NL-G-F/NL-G-F^ knock-in mice. CSF and cerebral Aβ42 were measured in 1 (n = 6), 2 (n = 12), 4 (n = 9), 6 (n = 13), and 9 (n = 12) months old App^NL-G-F/NL-G-F^ knock-in mice. (**a**) The concentration of Aβ42 in CSF was decreased, while (**b**) the concentration of Aβ42 in soluble protofibrils and (**c**, **d**) the burden of cortical Aβ42 immunoreactivity were increased, in an age-dependent manner. In a multiple regression model where both Aβ42 levels in brain soluble protofibrils and cortical immunoreactivity were included as predictors of the CSF Aβ42/Aβ40 ratio (**e**) or CSF Aβ42 (**f**), the Aβ42/Aβ40 ratio in CSF was independently associated with Aβ42 levels in soluble protofibrils but not cortical Aβ42 immunoreactivity. The Aβ42 concentration in brain soluble protofibrils fully mediated the effect of Aβ42 immunoreactivity on the CSF Aβ42/Aβ40 ratio (**g**) and CSF Aβ42 (**h**). Scale bars represent 100 µm. Mediation analysis alone does not establish causation. Bars represent partial R^*2*^ for each predictor in the model. The percentage within each bracket represents the ratio between the partial R^2^ and the total R^2^ of the model. Data are presented as median and IQR. Whiskers represent data within 1.5IQR of the lower and upper quartiles. For comparison between groups, statistical analyses were performed using the Kruskal-Wallis H test followed by the two-tailed Mann-Whitney U test for post hoc group comparisons (*p < 0.05, **p < 0.01, ***p < 0.001). The exact p-values are reported in the Source Data File for Extended Data Fig. 5. The results were not adjusted for multiple comparisons. Abbreviations: Aβ, amyloid beta; CSF, cerebrospinal fluid; IQR, interquartile range Source data

To confirm our findings in 5xFAD mice, we investigated the independent associations between the CSF Aβ_42_/Aβ_40_ ratio and measures of cerebral Aβ pathology, including Aβ_42_ in soluble protofibrils and plaques, in *App*^NL-G-F/NL-G-F^ knock-in mice^37^. However, we were unable to measure the concentrations of Aβ_40_ in brain soluble protofibrils in *App*^NL-G-F/NL-G-F^ knock-in mice with our current protocol because of the low concentrations of this Aβ peptide from the Beyreuther/Iberian mutation^37^. Like our results in 5xFAD mice, the concentration of Aβ_42_ in the CSF was reduced in an age-dependent manner (*H*(4) = 42.1, *P* < 0.001; Extended Data Fig. 5a) and coincided with increased concentrations of Aβ_42_ in soluble protofibrils (*H*(4) = 46.8, *P* < 0.001; Extended Data Fig. 5b), as well as increased cortical Aβ_42_ immunoreactivity in plaques (*H*(4) = 44.9, *P* < 0.001; Extended Data Fig. 5c,d). Importantly, in a multiple linear regression model in which both measures of Aβ pathology were included as predictors of the CSF Aβ_42_/Aβ_40_ ratio, we found that the CSF Aβ_42_/Aβ_40_ ratio was independently associated with the concentration of Aβ_42_ in soluble protofibrils (*β* = −1.24, *P* < 0.001) but not with Aβ_42_ immunoreactivity in plaques (*β* = 0.41, *P* = 0.055). Statistical mediation analysis adjusted for sex found that the Aβ_42_ concentration in protofibrils fully mediated (165%) the effect of Aβ_42_ immunoreactivity on the CSF Aβ_42_/Aβ_40_ ratio (Extended Data Fig. 5g). Similar results were found using CSF Aβ_42_ (Extended Data Fig. 5f,h and Supplementary Table 3).

### The Aβ_42_/Aβ_40_ ratio in protofibrils is the best predictor for the increase in CSF NfL and t-tau

In many studies, soluble Aβ oligomers and protofibrils have been implicated as upstream drivers of neuronal dysfunction and loss in AD^19^. However, it is not known to what degree these soluble aggregates relate to changes in established CSF biomarkers of neurodegeneration, including NfL and t-tau. Thus, building on our previously published findings showing that NfL and t-tau in CSF are increased in an age-dependent manner (Fig. 1b,c and Supplementary Table 1) relative to wild-type controls in 5xFAD mice^8,30^, the present investigation delved deeper into their association with different measures of Aβ pathology.

**Fig. 3:**
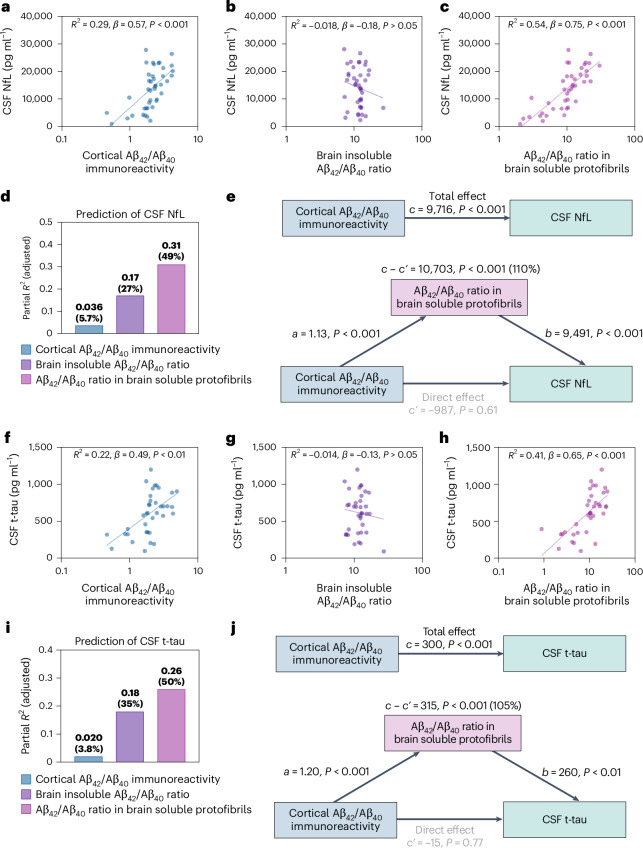
Associations between CSF biomarkers of neurodegeneration and measures of cerebral Aβ pathology. **a**–**c**, In simple linear regression models, CSF NfL was positively associated with cortical Aβ_42_/Aβ_40_ immunoreactivity (**a**) and the Aβ_42_/Aβ40 ratio in soluble protofibrils (**c**), while no association with the brain insoluble Aβ_42_/Aβ_40_ ratio was found (**b**). **d**, Multiple linear regression analysis showed that higher concentrations of CSF NfL were independently associated with higher Aβ_42_/Aβ_40_ ratios in soluble protofibrils and lower brain insoluble Aβ_42_/Aβ_40_ ratios. **e**, Mediation analysis revealed that the Aβ_42_/Aβ_40_ ratio in soluble protofibrils fully mediated the effect of cortical Aβ_42_/Aβ_40_ immunoreactivity on CSF NfL (110% mediation). **f**–**h**, Furthermore, in simple linear regression models, CSF t-tau was positively associated with cortical Aβ_42_/Aβ_40_ immunoreactivity (**f**) and the Aβ_42_/Aβ_40_ ratio in soluble protofibrils (**h**), while no association with the brain insoluble Aβ_42_/Aβ_40_ ratio was found (**g**). **i**, Multiple linear regression analysis showed that higher concentrations of CSF t-tau were independently associated with higher Aβ_42_/Aβ_40_ ratios in soluble protofibrils and lower brain insoluble Aβ_42_/Aβ_40_ ratios. The bars represent the partial *R*^2^ for each predictor in the model. The percentage within the parentheses represents the ratio between the partial and total *R*^2^ of the model. **j**, Mediation analysis revealed that the Aβ_42_/Aβ_40_ ratio in soluble protofibrils fully mediated the effect of cortical Aβ_42_/Aβ_40_ immunoreactivity on CSF t-tau (105% mediation). Mediation analysis alone does not establish causation. All analyses were adjusted for sex. The exact *P* values are reported in the source data file for Fig. 3. Source data

In the whole study population, simple linear regression models adjusted for sex showed that both the concentration of NfL and t-tau in CSF was positively correlated with cortical Aβ_42_/Aβ_40_ immunoreactivity (*R*^2^_(CSF NfL)_ = 0.29, *β*_(CSF NfL)_ = 0.57, *P*_(CSF NfL)_ < 0.001; *R*^2^_(CSF t-tau)_ = 0.22, *β*_(CSF t-tau)_ = 0.49, *P*_(CSF t-tau)_ < 0.01) (Fig. 3a,f) and soluble Aβ_42_/Aβ_40_ protofibrils (*R*^2^_(CSF NfL)_ = 0.54, *β*_(CSF NfL)_ = 0.75, *P*_(CSF NfL)_ < 0.001; *R*^2^_(CSF t-tau)_ = 0.41, *β*_(CSF t-tau)_ = 0.65, *P*_(CSF t-tau)_ < 0.001) (Fig. 3c,h), while no correlation with the Aβ_42_/Aβ_40_ ratio in formic acid-extracted Aβ was found (*R*^2^_(CSF NfL)_ = −0.018, *β*_(CSF NfL)_ = −0.18, *P*_(CSF NfL)_ > 0.05; *R*^2^_(CSF t-tau)_ = −0.014, *β*_(CSF t-tau)_ = −0.13, *P*_(CSF t-tau)_ > 0.001) (Fig. 3b,g). Comparison of the models revealed that the variance in the concentration of NfL and t-tau, respectively, was to a notably higher degree explained by soluble Aβ_42_/Aβ_40_ protofibrils than cortical Aβ_42_/Aβ_40_ immunoreactivity (Δ*R*_(CSF NfL)_ = 0.25, 95% CI = 0.11–0.42, *P* < 0.01; Δ*R*_(CSF t-tau)_ = 0.19, 95% CI = 0.035–0.35, *P* < 0.05).

We next performed multiple linear regression analyses to examine which of the measures of cerebral Aβ pathology are independently associated with increased concentrations of NfL and t-tau. All three measures of Aβ pathology were included as predictors and either CSF NfL or CSF t-tau as the outcome. We found that CSF NfL and t-tau were positively associated with soluble Aβ_42_/Aβ_40_ protofibrils (*β*_(CSF NfL)_ = 0.80, *P*_(CSF NfL)_ < 0.001; *β*_(CSF t-tau)_ = 0.64, *P*_(CSF t-tau)_ < 0.01) and negatively associated with the Aβ_42_/Aβ_40_ ratio in formic acid-extracted Aβ (*β*_(CSF NfL)_ = −0.36, *P*_(CSF NfL)_ < 0.01; *β*_(CSF t-tau)_ = −0.40, *P*_(CSF t-tau)_ < 0.01). No association with cortical Aβ_42_/Aβ_40_ immunoreactivity was found (*β*_(CSF NfL)_ = 0.23, *P*_(CSF NfL)_ = 0.25; *β*_(CSF t-tau)_ = 0.17, *P*_(CSF t-tau)_ = 0.43). The soluble Aβ_42_/Aβ_40_ ratio in protofibrils explained the maximum percentage of the variance in CSF NfL (partial *R*^2^ = 0.31, percentual *R*^2^ = 49%) and t-tau (partial *R*^2^ = 0.26, percentual *R*^2^ = 50%), respectively (Fig. 3d,i). When additionally including the overall brain soluble Aβ_42_/Aβ_40_ ratio in the models, the Aβ_42_/Aβ_40_ ratio in soluble protofibrils was the strongest independent predictor of high CSF NfL and t-tau concentrations, respectively (Supplementary Table 2).

**Extended Data Fig. 6:**
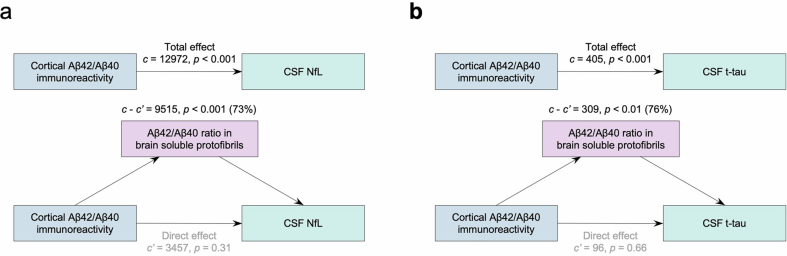
Mediation effect of the Aβ42/Aβ40 ratio in soluble protofibrils. Soluble Aβ42/Aβ40 protofibrils fully mediated the effect of cortical Aβ42/Aβ40 immunoreactivity on (**a**) CSF NfL, and (**b**) CSF t-tau when the models were adjusted for sex and brain insoluble Aβ42/Aβ40 ratios. Mediation analysis alone does not establish causation. Abbreviations: Aβ, amyloid beta; CSF, cerebrospinal fluid.

Statistical mediation analyses adjusted for sex showed that soluble Aβ_42_/Aβ_40_ protofibrils fully mediated the effect of cortical Aβ_42_/Aβ_40_ immunoreactivity on both CSF NfL (percentage mediation = 110%) and t-tau (percentage mediation = 105%) (Fig. 3e,j). Similar results were obtained when the models were additionally adjusted for the Aβ_42_/Aβ_40_ ratio in formic acid-extracted Aβ (Extended Data Fig. 6a,b). However, it is important to note that mediation analysis alone does not establish causation.

## Discussion

Together, these results suggest that reduced Aβ_42_/Aβ_40_ ratios in the CSF may, to a large degree, reflect enhanced formation of intermediate soluble Aβ protofibrils in the brain that are particularly enriched in Aβ_42_. Indeed, soluble oligomers and protofibrils of Aβ_42_ are especially sticky and bind to both cell membranes and receptors in the brain^19^. One could speculate that such binding properties of these Aβ_42_ species hinder the transport of Aβ_42_ into the CSF, which is supported by the fact that almost no Aβ oligomers and protofibrils are found in lumbar CSF, even in AD^38–40^.

Fibrillar Aβ dominates in the center of plaques^2^ and their accumulation during AD has previously been linked to reduced Aβ_42_/Aβ_40_ ratios in the CSF^6,7^. However, these findings are based on Aβ PET for visualization of fibrillar Aβ, which does not allow for the measurement of different Aβ peptides present in these insoluble deposits. This information might be of importance to better understand to what degree deposition of fibrillar Aβ contributes to reduced Aβ_42_/Aβ_40_ ratios in the CSF. In 5xFAD mice, we found that the Aβ_42_/Aβ_40_ ratio in fibrillar deposits was stable over time, which is congruent to a previous study finding only minor changes in the insoluble Aβ_42_/Aβ_40_ ratio over time in this mouse model^36^. Furthermore, this ratio had only a minor independent impact on the CSF Aβ_42_/Aβ_40_ ratio. This may suggest that the previously reported negative association with Aβ PET is secondary to other Aβ-related pathological processes, such as the formation of soluble Aβ protofibrils.

We found that the Aβ_42_/Aβ_40_ ratio in soluble protofibrils was the best predictor of CSF NfL and t-tau, which could imply that increased concentrations of NfL and t-tau in the CSF to some degree reflects neurotoxic processes mediated by soluble Aβ protofibrils enriched in Aβ_42_. Together, these findings may in part be related to the poor correlation between fibrillar Aβ burden in the brain, detected using amyloid PET, and clinical symptoms and neurodegeneration reported previously^41,42^.

Some limitations should be acknowledged in our study. First 5xFAD mice have an aggressive disease progression because of the overexpression of *APP* and *PS1*, which together harbor five mutations linked to familial AD. Although sample collection was initiated relatively early, at 2 months, deposition of Aβ both intracellularly and extracellularly had already started. Thus, although we recognize that information about the production rate of Aβ_42_ in relation to Aβ_40_ in the brain without Aβ plaques would have been an important addition to our models, estimation of these parameters was not possible from the collected samples. Second, with the extraction of Aβ from brain tissue samples, mechanical forces applied during homogenization and the centrifugation process have the potential to alter the native structure and properties of the peptide. Although our study used established protocols for Aβ extraction, it is important to acknowledge that the chosen methodology may have inherent limitations that could influence the aggregation state of the peptide during the sample preparation steps. Lastly, measurement of the concentrations of Aβ monomers and protofibrils in the interstitial fluid at different time points would have further contributed to our understanding of the associations between soluble Aβ oligomers and the investigated CSF biomarkers, and should be addressed in future studies.

Taken together, our findings suggest a mechanism where soluble Aβ protofibrils, rather than insoluble fibrillar Aβ deposits, may constitute a link between Aβ plaque pathology and reduced CSF Aβ_42_/Aβ_40_ ratios in AD. Consequently, the CSF Aβ_42_/Aβ_40_ ratio may be an indirect measure of the levels of Aβ_42_ oligomers and protofibrils in the brain and thereby provide different information compared to Aβ PET, which reflects the cored plaques in the brain. Furthermore, our results indicate that increased concentrations of the neurodegeneration markers NfL and t-tau in CSF may reflect neuronal injury mediated by soluble Aβ protofibrils. If replicated in future studies, these collective findings might have important implications for the interpretation of changes in the CSF Aβ_42_/Aβ_40_ ratio, as well as NfL and t-tau, in response to treatment in AD clinical trials. Normalization of the CSF Aβ_42_/Aβ_40_ ratio using therapies like lecanemab^43^ might indicate that such therapies not only remove insoluble Aβ fibrils (as revealed by amyloid PET), but also the brain levels of toxic Aβ oligomers and protofibrils.

## Methods

### Animals

The experimental procedures were carried out in accordance with Swedish animal research regulations and were approved by the committee of animal research at Lund University (ethical permit number no. 7482/2017). Animals were housed in groups of 2–6 mice per cage under a 12:12 h light–dark cycle with food and water provided ad libitum. The temperate was kept between 21 and 22 °C and humidity was kept at normal levels.

Male and female heterozygous 5xFAD mice (2–12 months; 2 months (*n* = 11), 4 months (*n* = 11), 6 months (*n* = 11), 12 months (*n* = 12)), originally obtained from The Jackson Laboratory, as well as male and female *App*^NL-G-F/NL-G-F^ knock-in mice (1–9 months; 1 month (*n* = 6), 2 months (*n* = 12), 4 months (*n* = 9), 6 months (*n* = 13), 9 months (*n* = 12)), were used in our experiments. Under the control of the mouse Thy1 promoter element, 5xFAD mice overexpress human APP (695) with the K670N/M671L (Swedish), I716V (Florida) and V717I (London) mutations together with human PS1 harboring the M146L and L286V mutations. Extracellular amyloid plaques start to accumulate in the deep cortical layers and subiculum around 2 months of age, spreading to other brain areas as the animal ages^36^. In *App*^NL-G-F/NL-G-F^ knock-in mice, the Aβ sequence of the endogenous *APP* gene has been humanized and three mutations associated with familial AD have been introduced: Swedish (KM670/671NL); Beyreuther/Iberian (I716F); and Arctic (E693G). This results in an age-dependent cerebral deposition of extracellular amyloid plaques starting from 2 months of age^37^.

### CSF and brain tissue collection

CSF was collected from the cisterna magna with a tapered-tip glass capillary as described previously^30^. All sample collection was performed between 9:00 and 13:00. After collection, the samples were immediately transferred to protein LoBind tubes, snap-frozen on dry ice and stored at −80 °C until analysis.

For the brain tissue collection, mice were transcardially perfused with ice-cold 0.1 M phosphate buffer (PB). The brain was removed and the cortex from the right hemisphere was dissected, collected in protein LoBind tubes, snap-frozen on dry ice and stored at −80 °C until analysis. The left hemisphere was fixed in 4% paraformaldehyde in 0.1 M PB, pH 7.4, for 48 h at 4 °C and then immersed in 30% sucrose solution for 48 h at 4 °C. Brains were serially cut into 30-μm-thick sagittal sections using a sliding microtome and collected in an antifreeze solution (30% sucrose and 30% ethylene glycol in PB) for storage at −20 °C.

### Biochemical analysis of the CSF

The concentration of human Aβ_42_ and Aβ_40_ and mouse t-tau in the collected CSF samples were measured using the Simoa Aβ_40_ and Aβ_42_ Advantage Kit (Quanterix) and the Simoa Mouse Tau Discovery Kit (Quanterix), respectively, on the Simoa HD-1 Analyzer (Quanterix) according to the instructions provided by the manufacturer. The concentrations of mouse NfL in the CSF were measured using an in-house Simoa NfL assay, in which the monoclonal antibodies and calibrators from the NF-light ELISA kit (UmanDiagnostics) were transferred onto the Simoa platform using a homebrew kit (Quanterix). The core domain of NfL, against which the antibodies were directed, is fully conserved between humans and mice^44^. The samples were run in singlicates and the respective measurements were performed in one round of experiments using the same batch of reagents.

### Immunohistochemistry, image acquisition and analysis

Thirty-micrometer-thick free-floating sagittal brain sections were washed three times for 10 min in Tris-buffered saline (TBS), incubated in 88% formic acid for 8 min, permeabilized three times for 10 min in TBS containing 0.25% Triton X-100 (TBSX) and blocked in TBSX containing 5% normal donkey serum (NDS) for 1 h at room temperature. The sections were then incubated with anti-Aβ_40_ (cat. no. 18580, IBL) or anti-Aβ_42_ (cat. no. 700254, Invitrogen) primary antibodies diluted 1:100 and 1:1,000, respectively, in TBSX containing 2.5% NDS overnight at 4 °C. The following day, sections were washed three times for 10 min in TBSX and incubated with the appropriate Alexa Fluor-conjugated secondary antibodies (Invitrogen) diluted 1:500 in TBSX containing 2.5% NDS for 2 h at room temperature. The sections were washed three times for 10 min in TBSX, mounted on glass slides and coverslipped using the ProLong Diamond Antifade Mountant (Invitrogen) according to the manufacturer’s recommendations.

Images of three different cortical regions in 4–5 sections per animal were captured using a ×10 objective lens on an Olympus IX70 fluorescence microscope equipped with a Hamamatsu ORCA-Flash4.0 LT+ digital complementary metal oxide semiconductor camera or a ZEISS Axio Imager M2 microscope with a ZEISS Axiocam 705 mono camera. The area (%) covered by Aβ_40_^+^ and Aβ_42_^+^ staining was quantified using the Fiji software by applying an automated local threshold that was maintained for all images analyzed. For each mouse, the total cortical area (%) covered by Aβ_40_^+^ and Aβ_42_^+^ staining was determined by calculating the average of the three cortical regions per section followed by the average of all captured sections. Images used to evaluate the localization of Aβ_40_^+^ and Aβ42^+^ staining in extracellular plaques were captured using a ×40 objective on a Leica SP8 laser scanning confocal microscope.

### Brain tissue homogenization

Cortex from the right hemisphere was homogenized at 10% (w/v) in TBS (50 mM Tris-HCl, 150 mM NaCl, pH 7.6) containing Halt Protease and Phosphatase Inhibitor Cocktail (Invitrogen) using the FastPrep-24 Classic bead beating grinder and lysis system (MP Biomedicals). The homogenized cortical brain tissue was aliquoted in protein LoBind tubes and stored at −80 °C until analysis.

### Biochemical analysis of Aβ_42_ and Aβ_40_ in insoluble brain tissue extracts

Prepared cortical brain tissue homogenates were thawed on ice and centrifuged at 14,000*g* for 30 min at 4 °C. The supernatant was collected as the TBS soluble fraction, aliquoted in protein LoBind tubes and stored at −80 °C until analysis. The remaining pellet was resuspended at 10% (v/w) in ice-cold 70% formic acid-containing Halt Protease and Phosphatase Inhibitor Cocktail, sonicated on ice six times for 10 s and centrifuged at 14,000*g* for 1 h at 4 °C. The supernatant was collected as the formic acid-soluble fraction, neutralized 1:20 in 1 M Tris-base at room temperature, aliquoted in protein LoBind tubes and stored at −80 °C until analysis.

The concentrations of Aβ_42_ and Aβ_40_ in the TBS-soluble and formic acid-soluble fractions prepared from the cortical brain tissue homogenates were measured using the MSD V-PLEX Aβ Peptide Panel 1 (6E10) Kit according to the manufacturer’s recommendations. All samples were measured in singlicates because this assay has consistently shown a low intraplate coefficient of variance in previous analyses.

### Biochemical analysis of Aβ protofibrils in soluble brain extracts

To measure Aβ protofibrils in soluble brain extracts, prepared homogenates were thawed on ice and centrifuged at 16,000*g* for 1 h at 4 °C. The supernatant, that is, the TBS-soluble fraction, was collected and the concentration of Aβ protofibrils was determined using an electrochemiluminescence-linked immunoassay. MULTI-ARRAY 96-well standard plates from Meso Scale Discovery were coated with the mouse monoclonal antibody mAb158, which has previously been shown to be selective for Aβ protofibrils^45–48^, followed by blocking in 1% Blocker A buffer (Meso Scale Discovery). Samples were diluted up to 1:640 times and loaded in duplicates onto coated 96-well plates, in which the Aβ protofibrils were allowed to bind to mAb158. Detection was performed using biotinylated mAb158 and Streptavidin SULFO-TAG (Meso Scale Discovery). The concentration of Aβ protofibrils in the samples was calculated from an Aβ protofibrils standard curve that was prepared in-house.

### Immunoprecipitation

To measure the concentrations of Aβ_42_ and Aβ_40_ in brain soluble Aβ protofibrils, prepared homogenates were thawed on ice and centrifuged at 16,000*g* for 1 h at 4 °C; then, the TBS-soluble fraction was collected. Immunoprecipitation (IP) was performed on the TBS-soluble brain tissue extracts using the mouse monoclonal antibody mAb158 to isolate the Aβ protofibrils. Initially, M-280 Tosylactivated Dynabeads (Invitrogen) were coupled to a mouse antimouse IgG2a monoclonal antibody (BD Pharmingen) according to the recommendations provided by the manufacturer. Briefly, coupling was performed overnight at 37 °C followed by blocking with 0.5% BSA in PBS for 1 h at 37 °C. Then, 20 μl TBS-soluble brain tissue extracts were diluted in 180 μl IP buffer (PBS containing 0.1% BSA and 0.5% Tween-20) containing the monoclonal antibody mAb158 (1.1 μg ml^−1^) and incubated for 1 h using a KingFisher Magnetic Particle Processor (Thermo Fisher Scientific). Then, 50 μl antibody-coupled Dynabeads were added to the samples and IP reactions were allowed to occur for 1 h. The Dynabeads were washed five times in IP buffer; then, the immunoprecipitated Aβ protofibrils were eluted and monomerized in 50 μl 1% SDS for 5 min at 95 °C.

The concentrations of Aβ_42_ and Aβ_40_ in the immunoprecipitated and monomerized Aβ protofibrils were measured in duplicate using the MSD V-PLEX Aβ Peptide Panel 1 (6E10) Kit according to the manufacturer’s recommendations.

### Statistics and reproducibility

The mice used in the present study were not randomized to experimental groups because the only variable was age. Data collection and analysis were performed blinded to the experimental groups. No statistical methods were used to predetermine sample size, but our sample sizes are similar to those reported in previous publications^30,32^. Data were not assumed to be normally distributed. The nonparametric Kruskal–Wallis test was performed to compare CSF biomarker concentrations and the measures of cerebral Aβ pathology between different age groups. If statistical significance was found, post hoc analysis for group comparisons between all age groups were done using a two-tailed Mann–Whitney *U*-test. No adjustments were made for multiple comparisons. Associations between each CSF biomarker (as the outcome variable) and the continuous measures of cerebral Aβ pathology (as the predictor variables) in the whole study population were tested in simple and multiple linear regression models adjusted for sex, in which the predictors were log_10_-transformed. For models with the same outcome variable, the adjusted *R*^2^ was compared between different regression models using bootstrapping (*n* = 5,000 iterations). Furthermore, mediation analyses with cortical Aβ_42_/Aβ_40_ immunoreactivity as the predictor, each CSF biomarker as the outcome and the Aβ_42_/Aβ_40_ ratio in brain soluble protofibrils as the mediator were conducted using bootstrapping, performed with the mediation package from R, to estimate the mediation effect. One mouse in which the Aβ_42_/Aβ_40_ ratio in insoluble fibrillar deposits was above three IQRs of the third quartile was excluded from the regression and mediation analyses. Statistical analyses were performed using SPSS v.27 and R v.4.1.0; the corresponding graphs were produced in Prism 9 (GraphPad Software).

### Reporting summary

Further information on research design is available in the Nature Portfolio Reporting Summary linked to this article.

## Supplementary information

Supplementary Information Supplementary Methods (MALDI mass spectrometry imaging and validation of the protofibril assay) and Tables 1–3.

Reporting Summary

## Source data

Source Data Data sheet containing the raw data for all measurements in 5xFAD mice.

Source Data Fig. 1 Exact *P* values for Fig. 1.

Source Data Fig. 2 Exact *P* values for Fig. 2.

Source Data Fig. 3 Exact *P* values for Fig. 3.

Source Data Extended Data Fig. 1 Exact *P* values for Extended Data Fig. 1.

Source Data Extended Data Fig. 2 Exact *P* values for Extended Data Fig. 2.

Source Data Extended Data Fig. 5 Raw data for all measurements in *App*^NL-G-F/NL-G-F^ mice.

Source Data Extended Data Fig. 5 Exact *P* values for Extended Data Fig. 5.

## Figures and Tables

**Extended Data Fig. 7:**
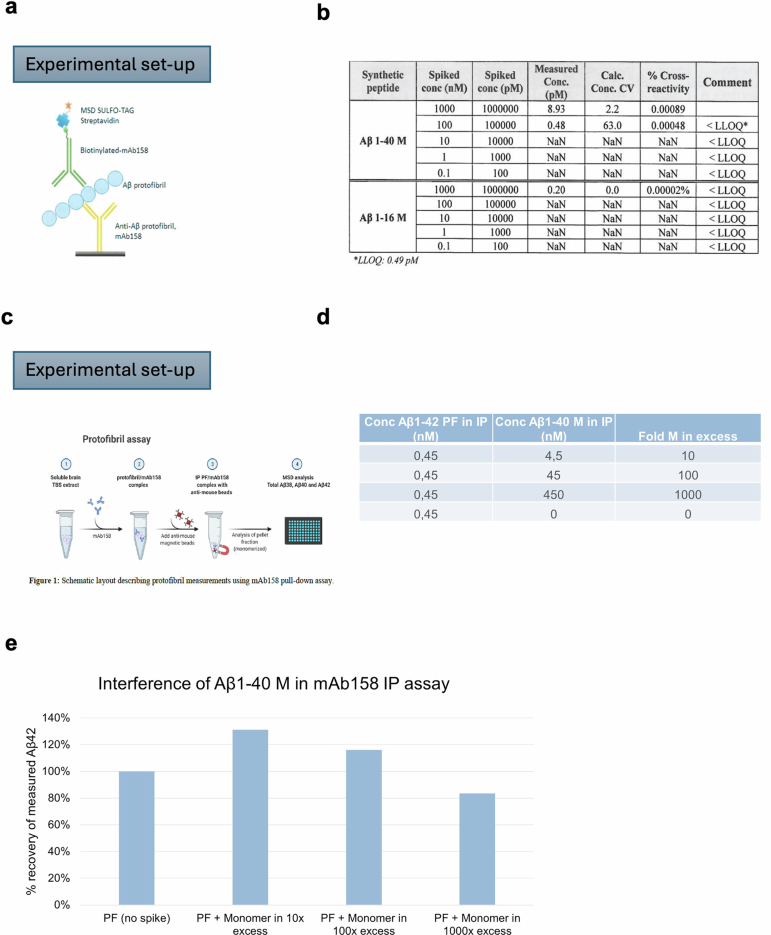
Validation of protofibril assay. No cross reactivity of mAb158 when spiked with synthetic Aβ1-16 or Aβ1-40 (**a, b**) in buffer. Introducing a 10-1000 excess of Aβ1-40 into brain homogenate did only minimally interfere at 1000-fold excess, while no interference was observed at 10-100-fold excess (**c, d, e**).

## Data Availability

All data and code are available in the article and Supplementary Information or will be shared upon reasonable request to the corresponding author from a qualified academic investigator. A response to the request shall be given within 2 weeks.
