# Supplementary material for: Soluble cerebral Aβ protofibrils link Aβ plaque pathology to changes in CSF Aβ42/Aβ40 ratios, neurofilament light and tau in Alzheimer’s disease model mice
Source: Nat Aging. 2025 Feb 12;5(3):366–75. doi: 10.1038/s43587-025-00810-8 (PMC11922755; doi:10.1038/s43587-025-00810-8)
Supplement: Supplementary file 1 — Supplementary Methods (MALDI mass spectrometry imaging and validation of the protofibril assay) and Tables 1–3. [file 43587_2025_810_MOESM1_ESM.pdf]

nature aging

Supplementary information

https://doi.org/10.1038/s43587-025-00810-8

# Soluble cerebral Aβ protofibrils link Aβ plaque pathology to changes in CSF $A\beta_{42}$/ $A\beta_{40}$ ratios, neurofilament light and tau in Alzheimer's disease model mice

In the format provided by the authors and unedited

# Supplementary information

## Table of Contents

***Supplementary information*** **........ 1**

**Supplementary methods ........ 2**

**MALDI mass spectrometry imaging** ........ 2

**Validation of protofibril assays** ........ 3

**Supplementary Tables ........ 4**

***Table S1*** ........ 4

***Table S2*** ........ 5

**Table S3** ........ 6

# Supplementary methods

## MALDI mass spectrometry imaging

Matrix assisted laser desorption/ionization (MALDI) mass spectrometry imaging (MSI) was performed to assess the relative deposition of Aβ42 and Aβ40 in cortical extracellular plaques. The brains from 4- and 6-months old male and female 5xFAD mice ($n$ = 3/timepoint) were collected within 3 min following decapitation and snap frozen on dry ice. Frozen brains were cut into 12 µm thick sagittal sections using a cryostat microtome (Leica CM 1520, Leica Biosystems, Nussloch, Germany) at −18°C, collected on indium tin oxide (ITO) conductive glass slides (Bruker Daltonics, Bremen, Germany), and stored at −80°C. Prior to analysis, tissue sections were thawed under vacuum for 1 hour.

Prior to matrix application, defrosted tissues were treated by sequential washes of 95% EtOH for 60 seconds, 70% EtOH for 30 seconds, Carnoy's solvent (60% EtOH, 30% chloroform, 10% acetic acid) for 90 seconds, 95% EtOH for 10 seconds, H2O with 0.2% TFA for 60 seconds, 95% EtOH for 15 seconds. For the washing procedures, the glass slides were placed upright in fresh solvents and let stand. For Aβ peptide signal enhancement, the tissue sections were hydrolyzed by exposure to vapor of concentrated formic acid for 25 min, as previously described[1]. MALDI matrix was applied using a TM sprayer (HTX Technologies, Carrboro, NC, USA) combined with a HPLC pump (Dionex P-580, Sunnyvale, CA, USA). Before spraying, the solvent pump was purged with 70% aqueous acetonitrile ($ACN_{aq}$) at 300µL/min for 5 min followed by manual rinse of matrix loading loop using a syringe. A matrix solution of 15 mg/mL 2′,5′-dihydroxyacetophenone (DHA, Sigma Aldrich) in 70% ACN/2%$CH_3COOH$/2%TFA was sprayed onto the tissue sections using the following instrumental parameters: nitrogen flow (10 psi), spray temperature (75°C), nozzle height (40 mm), eight passes with offsets and rotations, and spray velocity (1000 mm/min), and isocratic flow of 100 µL/min using 70% ACN as pushing solvent.

MALDI MSI was performed using a Bruker rapifleX TissueTyper TOF/TOF mass spectrometer (Bruker Daltonics) using the Flex Imaging software (v.4.0, Bruker). Peptide MSI data were acquired at 10 µm spatial resolution, at a laser pulse frequency of 10 kHz with 200 shots collected per pixel. Data were acquired in linear positive mode in the mass range of 1500–6000 Da (mass resolution: m/Δm=1000 (FWHM) at m/z 4515). Pre-acquisition calibration of the system was performed using a combination of peptide calibration standard II and protein calibration standard I, to ensure calibration over the entire range of potential Aβ species. Cortical plaque regions of interest (ROIs; 4-6 per mouse) were annotated in FlexImaging based on the Aβ1-42 signal ($M_{avg}$: 4515). Total ion current normalized average spectra of the annotated ROIs were exported as *.csv file followed by binning analysis. Here, all ROI data were imported into Origin (v 8.1 OriginLab, Northampton, MA, USA) and peaks and peak widths were detected on average spectra of each ROI using the implemented peak analyzer function. The determined bin borders for peak integration were exported as tab delimited text

file. The bin borders were used for area under curve peak integration within each bin (peak-bin) of all individual ROI average spectra using an in-house developed R script.

## Validation of protofibril assays

Cross reactivity test of synthetic Aβ1-40 monomers and Aβ1-16 peptide in sandwich mAb158 protofibril MSD assay (**Extended Data Fig. 7a**). Synthetic Aβ1-40 (American Peptide, Cat. No 62-0-78) and Aβ1-16 (Bachem, Cat no: H-2958) peptides were diluted in 1% Blocker A buffer (Meso Scale Discovery) to final concentrations of 1000, 100, 10, 1 and 0.1 nM. Samples were loaded in duplicates onto mAb158-coated 96-well plates and allowed to bind to mAb158. Detection was performed using biotinylated mAb158 and Streptavidin SULFO-TAG (Meso Scale Discovery). Obtained signals from synthetic Aβ1-40 and Aβ1-16 peptides were back-calculated to an Aβ protofibril standard curve with known concentration. Cross-reactivity was calculated from the percentage measured concentration to spiked known concentration (**Extended Data Fig. 7a**). Specificity of the mAb158 IP assay was evaluated by mAb158 binding to mixtures of Aβ1-42 synthetic protofibrils and Aβ1-40 monomers. Synthetic Aβ1-42 protofibrils were diluted in 1% Blocker A buffer (Meso Scale Discovery) to a final concentration of 0.45 nM in presence of Aβ1-40 monomers serially diluted ten-fold (450, 45, 4.5 nM) to generate protofibril to monomer ratio of 1:1000, 1:100, 1:10, respectively (**Extended Data Fig 7c, d**). Samples were incubated in presence of mAb158 (1.1 µg/ml) for 1 h using a KingFisher Magnetic Particle Processor) (Thermo Fisher Scientific), followed by an additional 1 h incubation after addition of 50 µl of M-280 Tosylactivated Dynabeads™ (Invitrogen) coupled to a mouse anti-mouse IgG2a monoclonal antibody (BD Pharmingen). The bound/immunoprecipitated fractions were eluted in 70% formic acid and neutralized in 1M Trizma+0.5M $Na_2HPO_4$ buffer prior to measurement of monomerized Aβ1-42 levels in the V-plex® Aβ peptide panel kit 1 (6E10). The recovery of protofibrils was calculated and presented as % recovery from non-spiked protofibril samples that was set to 100% (**Extended Data Fig. 7e**).

# References

1 Kakuda, N. *et al.* Distinct deposition of amyloid-beta species in brains with Alzheimer's disease pathology visualized with MALDI imaging mass spectrometry. *Acta Neuropathol Commun* **5**, 73, doi:10.1186/s40478-017-0477-x (2017).

# Supplementary Tables

*Table S1*: CSF Aβ42/Aβ40 ratio, NfL and t-tau in 5xFAD mice

| | **2 months** *n* = 11 | **4 months** *n* = 11 | **6 months** *n* = 11 | **12 months** *n* = 12 |
|---|---|---|---|---|
| **Sex (% female)** | 45.5 | 45.5 | 54.5 | 50.0 |
| **CSF Aβ42/Aβ40 ratio** | 1.84 (1.53-2.44) | 1.29 (1.17-1.34)[a] | 0.96 (0.77-1.18)[a, d] | 0.59 (0.55-0.68)[a, b, c] |
| **CSF Aβ42 (pg/ml)** | 15327 (14006-19535) | 11096 (9052-12310)[a] | 7359 (6814-7823)[a, b] | 4592 (4479-4771)[a, b, c] |
| **CSF Aβ40 (pg/ml)** | 8381 (5719-12900) | 9067 (7599-9446) | 6964 (6340-7700) | 7603 (6669-8228) |
| **CSF t-tau (pg/ml)** | 318 (184-353) | 608 (445-807)[a] | 715 (586-961)[e] | 726 (695-924)[a] |
| **CSF NfL (pg/ml)** | 4186 (2744-7730) | 9690 (6770-13643)[f] | 15306 (13857-18425)[a, d] | 22252 (19020-23389)[a, b, g] |

CSF Aβ42/Aβ40 ratios, NfL and t-tau were measured in 2 ($n$=11), 4 ($n$ = 11), 6 ($n$ = 11), and 12 ($n$ = 12) months old 5xFAD mice. Data are presented as median and IQR. Kruskal-Wallis H test was performed to study differences between the groups. If statistically significant differences were found, *post hoc* analysis for group comparisons were performed using the two tailed Mann-Whitney U test ([a] $p < 0.001$ vs. 2 months, [b] $p < 0.001$ vs. 4 months, [c] $p < 0.001$ vs. 6 months, [d] $p < 0.05$ vs. 4 months, [e] $p < 0.01$ vs 2 months, [f] $p < 0.05$ vs. 2 months, [g] $p < 0.01$ vs. 6 months). The exact p-values are reported in the Source Data File for Figure 1 and Extended Data figure 1. No adjustments were made for multiple comparisons. Abbreviations: Aβ, amyloid beta; CSF, cerebrospinal fluid; IQR, interquartile range; NfL, neurofilament light; t-tau, total tau.

*Table S2*: Prediction of the CSF Aβ42/Aβ40 ratio, NfL, and t-tau in 5xFAD mice

| | CSF Aβ42/Aβ40 ratio | | | CSF NfL | | | CSF t-tau | | |
|---|---|---|---|---|---|---|---|---|---|
| | **Partial $R^2$** | **$\beta$** | **$p$-value** | **Partial $R^2$** | **$\beta$** | **$p$-value** | **Partial $R^2$** | **$\beta$** | **$p$-value** |
| **Cortical Aβ42/Aβ40 immunoreactivity** | 0 | 0.05 | 0.789 | 0.01 | 0.14 | 0.55 | 0 | 0.06 | 0.83 |
| **Brain insoluble Aβ42/Aβ40 ratio** | 0 | 0.15 | 0.129 | 0.14 | –0.34 | 0.005* | 0.14 | –0.36 | 0.015* |
| **Soluble Aβ42/Aβ40 protofibrils** | 0.23 | –0.60 | 0.002* | 0.12 | 0.63 | 0.037* | 0.13 | 0.54 | 0.039* |
| **Overall brain soluble Aβ42/Aβ40 ratio** | 0.05 | –0.31 | 0.161 | 0.02 | 0.23 | 0.43 | 0.01 | 0.20 | 0.52 |

Multiple linear regression model including cortical Aβ42/Aβ40 immunoreactivity, FA-extracted brain insoluble Aβ42/Aβ40 ratio, soluble Aβ42/Aβ40 protofibrils, and the overall soluble Aβ42/Aβ40 ratio as predictors of the CSF Aβ42/Aβ40 ratio, NfL, and t-tau, respectively (*p<0.05). *Note that the associations between CSF biomarkers and brain insoluble Aβ42/Aβ40 ratio are positive, but the ones with soluble Aβ42/Aβ40 protofibrils are negative as expected (where more protofibrils in the brain are associated with lower CSF Aβ42/Aβ40 values).*

**Table S3**: Prediction of CSF Aβ42/Aβ40 ratio and CSF Aβ42 in $APP^{NL\text{-}G\text{-}F/NL\text{-}G\text{-}F}$ knock in mice

| | **CSF Aβ42/Aβ40 ratio** | | | **CSF Aβ42** | | |
|---|---|---|---|---|---|---|
| | **Partial $R^2$** | ***β*** | ***p*-value** | **Partial $R^2$** | ***β*** | ***p*-value** |
| **Cortical Aβ42 immunoreactivity** | 0.074 | 0.41 | 0.055 | $2.2 * 10^{-8}$ | -0.00025 | 0.999 |
| **Soluble Aβ42 protofibrils** | 0.41 | -1.24 | $3.92*10^{-7}$* | 0.19 | -0.82 | 0.001* |

Multiple linear regression model including cortical Aβ42 immunoreactivity and soluble Aβ42 protofibrils as predictors of the CSF Aβ42/Aβ40 ratio and CSF Aβ42 respectively (*$p<0.05$).
