# Supplementary material for: Soluble cerebral Aβ protofibrils link Aβ plaque pathology to changes in CSF Aβ42/Aβ40 ratios, neurofilament light and tau in Alzheimer’s disease model mice
Source: Nat Aging. 2025 Feb 12;5(3):366–75. doi: 10.1038/s43587-025-00810-8 (PMC11922755; doi:10.1038/s43587-025-00810-8)
Supplement: Supplementary file 2 — Reporting Summary [file 43587_2025_810_MOESM2_ESM.pdf]

nature portfolio

Corresponding author(s): Oskar Hansson

Last updated by author(s): Nov 1, 2024

Reporting Summary

Nature Portfolio wishes to improve the reproducibility of the work that we publish. This form provides structure for consistency and transparency in reporting. For further information on Nature Portfolio policies, see our Editorial Policies and the Editorial Policy Checklist.

Statistics

For all statistical analyses, confirm that the following items are present in the figure legend, table legend, main text, or Methods section.

n/a	Confirmed	
☐	☒	The exact sample size (*n*) for each experimental group/condition, given as a discrete number and unit of measurement
☐	☒	A statement on whether measurements were taken from distinct samples or whether the same sample was measured repeatedly
☐	☒	The statistical test(s) used AND whether they are one- or two-sided<br>*Only common tests should be described solely by name; describe more complex techniques in the Methods section.*
☐	☒	A description of all covariates tested
☐	☒	A description of any assumptions or corrections, such as tests of normality and adjustment for multiple comparisons
☐	☒	A full description of the statistical parameters including central tendency (e.g. means) or other basic estimates (e.g. regression coefficient) AND variation (e.g. standard deviation) or associated estimates of uncertainty (e.g. confidence intervals)
☐	☒	For null hypothesis testing, the test statistic (e.g. *F*, *t*, *r*) with confidence intervals, effect sizes, degrees of freedom and *P* value noted<br>*Give P values as exact values whenever suitable.*
☒	☐	For Bayesian analysis, information on the choice of priors and Markov chain Monte Carlo settings
☒	☐	For hierarchical and complex designs, identification of the appropriate level for tests and full reporting of outcomes
☒	☐	Estimates of effect sizes (e.g. Cohen's *d*, Pearson's *r*), indicating how they were calculated

Our web collection on statistics for biologists contains articles on many of the points above.

Software and code

Policy information about availability of computer code

Data collection	No software was used for data collection.
Data analysis	R (v. 4.1.0) , including the mediation package, and IBM SPSS statistics 27 were used for data analyses as described in the manuscript. Graphs were created using Graphpad Prism 9. Microsoft Excel (Version 16.90) was used.

For manuscripts utilizing custom algorithms or software that are central to the research but not yet described in published literature, software must be made available to editors and reviewers. We strongly encourage code deposition in a community repository (e.g. GitHub). See the Nature Portfolio guidelines for submitting code & software for further information.

Data

Policy information about availability of data

All manuscripts must include a data availability statement. This statement should provide the following information, where applicable:

- Accession codes, unique identifiers, or web links for publicly available datasets
- A description of any restrictions on data availability
- For clinical datasets or third party data, please ensure that the statement adheres to our policy

All data and code are available within the article and supplementary information or will be shared by request to the corresponding author from a qualified academic investigator. A response to the request shall be given within two weeks of time.

Research involving human participants, their data, or biological material

Policy information about studies with human participants or human data. See also policy information about sex, gender (identity/presentation), and sexual orientation and race, ethnicity and racism.

Reporting on sex and gender	NA
Reporting on race, ethnicity, or other socially relevant groupings	NA
Population characteristics	NA
Recruitment	NA
Ethics oversight	NA

Note that full information on the approval of the study protocol must also be provided in the manuscript.

Field-specific reporting

Please select the one below that is the best fit for your research. If you are not sure, read the appropriate sections before making your selection.

☒ Life sciences ☐ Behavioural & social sciences ☐ Ecological, evolutionary & environmental sciences

For a reference copy of the document with all sections, see nature.com/documents/nr-reporting-summary-flat.pdf

Life sciences study design

All studies must disclose on these points even when the disclosure is negative.

Sample size	No statistical method was used to predetermine sample size. Sample size was based off previous literature (Maia et al 2013 ,DOI: 10.1126/scitranslmed.3006446, Andersson et al 2020 DOI: 10.1016/j.neurobiolaging.2020.07.018.) and on past experience with the particular animal models. The exact sample sizes are reported in the figures as well as supplementary table 1 and Statistics and Reproducibility section in the methods.
Data exclusions	As stated in the manuscript:, one mouse in which the Aβ42/Aβ40 ratio in insoluble fibrillar deposits was above 3 interquartile ranges of the third quartile was excluded from regression and mediation analyses.
Replication	We replicated the majority of our results from the 5xFAD mouse model in the APP NL-G-F mouse model. All attempts at replication were succesful. As stated in the manuscript, the low levels of Aβ-40 peptide as a result of the Beyreuther/Iberian mutation in the APP NL-G-F mice, prevented us from measuring Aβ-40 in brain soluble protofibrils in these mice. None of the other experiments were replicated.
Randomization	NA as our groups of mice were only seperated by age.
Blinding	The investigators were blinded as each mouse was only given a numerical identifier during data collection and analyses.

Reporting for specific materials, systems and methods

We require information from authors about some types of materials, experimental systems and methods used in many studies. Here, indicate whether each material, system or method listed is relevant to your study. If you are not sure if a list item applies to your research, read the appropriate section before selecting a response.

Materials & experimental systems

n/a	Involved in the study	
☐	☒	Antibodies
☒	☐	Eukaryotic cell lines
☒	☐	Palaeontology and archaeology
☐	☒	Animals and other organisms
☒	☐	Clinical data
☒	☐	Dual use research of concern
☒	☐	Plants

Methods

n/a	Involved in the study	
☒	☐	ChIP-seq
☒	☐	Flow cytometry
☒	☐	MRI-based neuroimaging

Antibodies

Antibodies used	The following antibodies were used: anti-Aβ40 (IBL, #18580), anti-Aβ42 (Invitrogen, #700254)
Validation	Aβ40 (IBL, #18580), has been validated using western blot and immunohistochemistry and tested for cross reactivity with other forms of Aβ. Anti-Aβ42 (Invitrogen, #700254) has been validated with immunohistochemistry and tested for cross reactivity with other fMorms of Aβ in sandwich Elisa. For more detail see the manufacturers website.<br>mAb158 is the murine version of Lecanemab, and has been validated in several papers to bind specifically to amyloid protofibrils. Tucker et al 2015, (DOI: 10.3233/JAD-140741, Johannesson et al 2024 (DOI : 10.1016/j.mcn.2024.103949)<br>mouse anti-mouse IgG2a monoclonal antibody (BD Pharmingen, https://www.bdbiosciences.com/en-ca/products/reagents/immunoassay-reagents/elisa/purified-mouse-anti-mouse-igg2a-a.553501) Has been validated and routinely used for this method by Bioarctic

Animals and other research organisms

Policy information about studies involving animals; ARRIVE guidelines recommended for reporting animal research, and Sex and Gender in Research

Laboratory animals	Male and female heterozygus 5xFAD mice were used between the ages of 2-12 months. These mice were obtained from Jackson Laboratory.<br>Male and female APP NL-G-F/NL-G-F knock in mice were used between the ages of 1-9 months. These were originally obtained from Takaomi C Saido<br>Animals were housed in groups of 2-6 mice per cage under a 12:12 h light/dark cycle with food and water provided ad libitum. The temperate was controlled between 21 and 22 °C and humidity was kept at normal levels.
Wild animals	No wild animals were used in the study.
Reporting on sex	A 50% male to female ratio was aimed for the study. Sex was used as a covariate in the analyses.
Field-collected samples	No field samples were collected in the study
Ethics oversight	As stated in the manuscript: The experimental procedures were carried out in accordance with Swedish animal research regulations and were approved by the committee of animal research at Lund University (ethical permit number: 7482/2017).

Note that full information on the approval of the study protocol must also be provided in the manuscript.

Plants

Seed stocks	NA
Novel plant genotypes	NA
Authentication	NA
